# The effect of kangaroo mother care on physiological parameters of premature infants in Hamadan City, Iran

**DOI:** 10.11604/pamj.2018.30.89.14428

**Published:** 2018-05-31

**Authors:** Parisa Parsa, Simin Karimi, Behnaz Basiri, Godratalah Roshanaei

**Affiliations:** 1 Chronic Diseases (Home Care) Research Center, Hamadan University of Medical Sciences, Hamadan, Iran; 2 Student Research Committee,Hamadan University of Medical Sciences, Hamadan, Iran; 3 Faculty of Medicine, Hamadan University of Medical Sciences, Hamadan, Iran; 4 Faculty of Public Health, Hamadan University of Medical Sciences, Hamadan, Iran

**Keywords:** Kangaroo care, physiological indices, premature infants

## Abstract

**Introduction:**

Breast-feeding and being with mother have positive effects on the preterm infant's health status. Therefore, this study was conducted to evaluate the effect of Kangaroo Mother Care (KMC) on physiological parameters of premature infants in Fatemiyeh Hospital in Hamadan in 2016.

**Methods:**

This was a quasi-experimental study. One hundred newborns who were admitted to in neonatal intensive care unit of Fatemiyeh Hospital in Hamadan city, Iran were selected by convenience sampling. They were randomly divided into two groups (experimental group, n = 50 and control group, n = 50). In the experimental group, newborns were taken daily KMC for an hour during 7 days. In the control group, routine care was performed in the incubator. The data gathering tool was questionnaire of infants and mother characteristics, checklists of vital signs and oxygen saturation. Data analysis was performed by SPSS 19 software using descriptive and inferential statistics (Independent t -test, Paired t-test, Chi-square, ANOVA).

**Results:**

Before intervention, there was no significant difference between the physiological parameters of the infants (heart rate, respiratory rate, arterial blood oxygen saturation and temperature) in experimental and control groups. However, after intervention, there was a significant difference between the two groups in terms of physiological indices (p < 0.001).

**Conclusion:**

The findings of this study indicate the effect of KMC on enhancement of physiological indices. Therefore, it is recommended that KMC is taken as one of the routine care of premature infants.

## Introduction

Neonates born before 37 completed weeks of pregnancy are called premature infants. The birth of premature infants is associated with several problems, such as frequent hospital admissions, infections, apnea and others [1]. Despite the comprehensive efforts to prevent premature delivery and birth of premature infants, the birth rates of such infants are high due to some medical problems, social status and infertility treatment [2,3]. In the United States, there are about 250,000 premature and low birth weight infants each year, accounting for 8.8% of births [4]. In Iran, 5000 neonates are born daily, about 12% of them are underweight [5]. Therefore, care for such infants is a burden on community health systems. Most common method for care of premature infants is incubator method. In this way, the infant undergoes a special care in a glass device, apart from a mother. Meanwhile, in alternative method as known as Kangaroo Mother Care (KMC), the baby is placed between the mother's breasts in an upright position. Mother secures him with the binder. The baby's head, turned to one side, is in a slightly extended position. This position keeps the airway open and allows eye-to-eye contact between the mother and the baby. The hips should be flexed and extended in a “frog” position; the arms should also be flexed. The tight part of the cloth is over the baby's chest. Baby's abdomen should not be constricted and should be somewhere at the level of the mother's epigastrium. This way baby has enough room for abdominal breathing. Mother's breathing stimulates the baby [6]. KMC was initiated in 1978 in Colombia as a way to offset the lack of human resources and other infant care facilities [4]. The high prevalence of premature infants, the lack of specialized care equipment and the high mortality rate of premature infants are among the reasons for the use of KMC for premature infants [7]. Various studies have shown that KMC has had favorable results for neonates and mothers, which includes: favorable effects on heart rate, oxygen saturation and respiratory rate [8], maintaining body temperature and sleeping of the infant [9], positive effect on mental and cognitive development, better performance in physical tests during early childhood [10], helping to increase mother's emotional feelings toward the newborn [11,12], positive effect on family attachment [13,14], and confidence in mother-child care [15]. It may also affect the subsequent outcomes and long-term welfare of the mothers and their babies for reducing risky behaviors later in her life [16]. Since there has been no research on the effect of KMC on the physiological indices of premature infants in Hamadan province, this study investigated the effect of KMC on the physiological parameters such as heart rate, respiratory rate, arterial oxygen saturation and body temperature in premature neonates admitted in NICU of Fatemiyeh Hospital, Hamadan city, Iran.

## Methods

The present study was a quasi-experimental study in premature neonates admitted to the Neonatal Intensive Care Unit (NICU) of Fatemieh Hospital in Hamadan. The study was conducted from February to September, 2016. Sample size was estimated according to the preliminary study and the following formula with considering 95% confidence level, power of 90% and 10% of sample drop. In the formula: Z is standard normal deviation set at 95% level; µ is assumed population mean and σ is the estimated standard deviation. Therefore, 50 neonate in each group were obtained. In total, two groups of 100 neonates were considered.

$$n=\frac{{\left(Z_{1-\frac{\alpha }{2}}+Z_{1-\beta }\right)}^{2}\left(\sigma_{1}^{2}+\sigma_{2}^{2}\right)}{{\left(\mu_{2}-\mu_{1}\right)}^{2}}$$

Inclusion criteria were: infant weight at birth less than 2500 grams, neonatal birth age of 34-36 weeks of gestation, neonates stable enough to leave the incubator and be with the mother (a pediatrics allows the baby to leave the incubator), and the newborn has not undergone surgery. Exclusion criteria were: neonates too sick to participate in the study, neonates on mechanical ventilation, maternal illness or complications preventing her from caring her baby, decline parental consent before or during the study. The newborns who met the inclusion criteria were selected by convenience sampling to complete the sample size (100 neonates) . Before the beginning of the study, informed consent was obtained from the infant's parents. Then, the infants were randomly divided into experimental and control groups. The two experimental and control groups were homogeneous in terms of infant's weight and gestational age. Before intervention, demographic data were collected by a questionnaire and neonates of both groups were evaluated for body temperature, oxygen saturation, heart rate, respiratory rate. The questionnaire consisted of characteristics of the mother and her neonate including maternal complications during pregnancy and breastfeeding status. Physiological indicators (heart rate, respiratory rate, oxygen saturation, temperature) were measured and recorded on the first, third, fifth and seventh days of KMC. In the experimental group, first, mothers got bathing, wearing a special KMC blanket, and perform KMC. With the help of nursing staff, neonate was placed between mothers' breasts . KMC care conducted for an hour per day for seven days. Room temperature was 26-29°C. In the control group, conventional care (incubator) was performed. For both groups, the physiological parameters of the infant were measured 3 times in each KMC practice ((before, 15 minutes after initiation KMC (median) and after completed KMC)) and recorded in the checklist. At the end, changes in physiological indices of premature infants were compared in both groups. Data were analyzed using SPSS version 19, independent t-test, Paired t-test, Chi-square test. P- Value less than 0.05 was considered as significant level.

## Results

Demographic characteristics of subjects are presented in Table 1. Based on the results of the study, the data were homogeneous in terms of mothers' age, fathers' age, parents' employment status and education levels, and family income (p>0.05). There was no significant difference in the rate of infants' heart rate in two groups before intervention (P > 0.05). After KMC, significant change were observed in heart rate of experimental group while no significant change was seen in the control group (Table 2). The respiratory rate of infants in the experimental and control groups was not significantly different before intervention (P > 0.05). Significant change was observed in respiratory rate of experimental group after KMC intervention, while no significant change was seen in the control group (Table 3). Before KMC, intervention, the percentage of arterial blood oxygen saturation in the experimental and control groups was not different (p > 0.05). After KMC, significant change was observed in arterial blood oxygen saturation of experimental group after KMC, while no significant change was seen in the control group (Table 4). The axillary temperature of the neonates in two groups did not show a significant difference before KMC (P > 0.05). After KMC, significant change was observed in axillary temperature of experimental group, while no significant change was seen in the control group (p < 0.01) (Table 5).

**Table 1:** Distribution of demographic characteristics of experimental and control groups

Characteristics	Experimental		Control		Statistics
	N	%	N	%	P
**Age of Mothers (year)**					0.510
<20	4	8	2	4	
21-30	31	62	36	72	
>30	15	30	12	24	
**Age of Fathers**					0.898
21-30	31	62	32	64	
31-40	18	36	16	32	
>40	1	2	2	4	
**Mothers' Education**					0.440
Elementary	1	2	6	12	
Secondory	17	34	17	34	
Trtiry	32	64	27	54	
**Fathers' Education Level**					P=0.438
Elementary	0	0	1	2	
Secondory	8	16	10	20	
Trtiry	42	84	39	78	
**Mothers' Occupation Status**					P=0.840
Employed	2	4	1	2	
Unemployed	48	96	49	98	
**Fathers' Occupation Status**					P=0.710
Employed	49	98	50	100	
Unemploye	1	2	0	0	
**Family income ($)**					P=0.158
<500	31	62	33	66	
>500	19	38	14	28	

**Table 2:** Comparison of physiological index of heart rate in the experimental and control groups

Heart Rate	Experimental		Control		t	p
	Mean	SD	Mean	SD		
**Day 1**						
Before	166.82	12.761	164.00	11.185	1.175	0.243
Middle	146.46	6.600	165.48	10.298	10.995	<0.001
After	149.10	7.731	165.82	9.275	9.792	<0.001
**Day 3**						
Before	167.98	12.180	166.54	11.075	0.619	0.538
Middle	145.32	7.144	167.32	10.129	12.551	<0.001
After	148.20	7.706	167.08	9.722	10.761	<0.001
**Day 5**						
Before	166.92	11.362	166.08	9.044	0.409	0.683
Middle	144.36	7.053	166.16	8.863	13.610	<0.001
After	147.86	7.532	165.84	9.416	10.544	<0.001
**Day 7**						
Before	165.74	10.532	165.88	8.571	0.073	0.942
Middle	144.24	6.313	166.06	8.355	14.733	<0.001
After	147.84	6.313	166.44	8.841	12.106	<0.001

**Table 3:** Comparison of physiological index of respiratory rate in the experimental and control groups

Respiratory Rate	Experimental		Control		t	p
	Mean	SD	Mean	SD		
**Day 1**						
Before	66.36	8.796	65.04	8.473	0.764	0.447
Middle	47.10	5.100	65.98	6.570	-16.052	<0.001
After	49.24	5.572	66.34	6.638	-13.952	<0.001
**Day 3**						
Before	66.70	6.816	66.80	8.091	-0.067	0.947
Middle	46.32	4.851	66.88	7.079	-16.942	<0.001
After	49.30	5.418	67.12	7.356	-13.792	<0.001
**Day 5**						
Before	65.38	8.664	66.74	6.892	-0.869	0.387
Middle	46.24	5.200	66.96	6.168	-18.161	<0.001
After	49.08	5.264	67.14	6.220	-15.672	<0.001
**Day 7**						
Before	64.80	6.575	65.94	6.281	-0.887	0.377
Middle	45.92	4.856	66.56	6.072	-18.772	<0.001
After	48.52	4.846	67.40	6.955	-15.750	<0.001

**Table 4:** Comparison of physiological index of arterial oxygen saturation in the test and control group

Arterial oxygen saturation%	Experimental		Control		t	p
	Mean	SD	Mean	SD		
**Day 1**						
Before	87.20	2.619	87.84	3.507	-1.034	0.304
Middle	97.02	1.407	87.80	2.799	20.809	<0.001
After	95.30	1.607	87.88	2.745	16.495	<0.001
**Day 3**						
Before	87.72	2.119	88.20	2.983	-0.928	0.356
Middle	97.70	0.931	87.88	2.723	24.130	<0.001
After	95.92	1.353	87.60	2.571	20.248	<0.001
**Day 5**						
Before	88.04	1.577	88.06	2.428	-0.049	0.961
Middle	98.16	1.095	87.88	2.496	26.670	<0.001
After	96.20	1.512	87.64	2.328	21.807	<0.001
**Day 7**						
Before	89.02	2.085	88.34	2.536	1.465	0.146
Middle	98.18	1.380	87.78	2.452	26.135	<0.001
After	96.56	2.196	87.70	2.533	18.686	<0.001

**Table 5:** Comparison of physiological index of temperature in the test and control group

Temperature	Experimental		Control		t	p
	Mean	SD	Mean	SD		
**Day1**						
Before	36.424	0.2559	36.324	0.2536	1.967	0.052
Middle	36.908	0.1243	36.314	0.2222	16.495	<0.001
After	36.786	0.1400	36.318	0.2371	12.020	<0.001
**Day 3**						
Before	36.443	0.2142	36.294	0.2208	3.419	0.001
Middle	36.898	0.1708	36.300	0.2138	15.451	<0.001
After	36.807	0.1760	36.292	0.1978	13.768	<0.001
**Day 5**						
Before	36.470	0.2159	36.294	0.2280	3.963	<0.001
Middle	36.944	0.1327	36.274	0.2008	19.681	<0.001
After	36.876	0.1492	36.286	0.2010	16.657	<0.001
**Day 7**						
Before	36.503	0.2542	36.348	0.2043	3.355	0.001
Middle	36.964	0.1782	36.282	0.2173	17.162	<0.001
After	36.866	0.2026	36.318	0.2106	13.258	<0.001

## Discussion

According to results after KMC, significant differences in physiological indices were observed between the experimental and control groups. In the study of Nourian et al. who compared the effect of KMC and routine care methods on physiological criteria in low birth weight infants, no significant differences were observed between two groups during intervention (P > 0.05). However, significant differences were seen between two groups in terms of heart rate, oxygen saturation and respiratory rate 5 minutes after intervention (p < 0.05). Infants' temperature had no changes during this study. The results showed that KMC care is effective on the sustainability of physiological parameters during care. Therefore, caregivers should take kangaroo mother care for mothers and infants [8].

In the present study, there was a significant difference between the two groups in terms of physiological criteria not only after KMC care (P < 0.001), but also during kangaroo care (P < 0.001). Similarly, Keshavarz and colleagues studied the effect of KMC on infants' physiological parameters, infants' crying, and mothers' pain after cesarean section. One hundred-sixty mothers and infants were randomly assigned into two groups of KMC and routine care. The two groups were not different in terms of mother and infant charactristics. In the Kangaroo care group, the average of the infant's temperature at half an hour (36.8 vs. 36.6°C, P > 0.05) and one hour (36.9 vs. 36.6°C, P < 0.001) after skin to skin contact were more than the control group. Also, in kangaroo care group the score of mothers' pain after cesarean section (6 versus 7.8, P < 0.001), the frequency of infants' crying (5.6 vs. 12.3 times, P < 0.05) were lower than the control group. Overall, mothers were satisfied with Kangaroo care [17]. In contrast of our results, Jafari and colleagues studied KMC in weight gain, duration of hospitalization, and body temperature of premature infants. The findings showed no significant difference in term of infants' temperature between the two groups after KMC (p>0.05) [18].

In a study by Basiri et al., the effect of duration of KMC on the neonatal growth of low birth weight infants was studied. One hundred-fifteen LBW neonates were randomly divided into two groups, the first group received the maximum of 4 hours KMC per a day and the second group got more than 4 hours KMC per day. In the Kangaroo care more than 4 hours, the mean and standard deviation of oxygen saturation was higher than a group with less than 4 hours Kangaroo care (P > 0.05). But, no significant difference was found between the two groups in terms of body temperature. Infants' growth and physiological criteria were better in the group with more than 4 hours KMC compared to less than 4 hours KMC [19]. Therefore, it is essential that the strategies of increasing the duration of kangaroo care be taken into consideration by policy makers and healthcare providers. In the present study, the duration of kangaroo care was not considered, the results for oxygen saturation were in line with a Basiri study. It is suggested that, the positive effects of kangaroo care and the safety of this method taking into account and KMC is used more widely.

## Conclusion

Kangaroo Mother Care improves physiological indices in normal levels, thus it might positively influence the premature infant's physical health. Further study is needed to determine the long-term outcomes of KMC in low birth weight and premature infants.

### What is known about this topic

- Prematurity is associated with several problems for newborn and their family;
- Care for premature infants is a burden on community health systems;
- Prevalence of premature newborn varies among countries.

### What this study adds

- Kangaroo Mother Care is a good way to take care of premature and low birth weight infants;
- The Kangaroo Mother Care improves physiological parameters of premature newborns;
- To improve health status of premature newborn, increasing the duration of Kangaroo Mother Care can be taken into consideration by policy makers and healthcare providers.
